# Assessment of Ecological and Human Health Risks of Heavy Metal Contamination in Agriculture Soils Disturbed by Pipeline Construction

**DOI:** 10.3390/ijerph110302504

**Published:** 2014-02-28

**Authors:** Peng Shi, Jun Xiao, Yafeng Wang, Liding Chen

**Affiliations:** 1 State Key Laboratory of Urban and Regional Ecology, Research Center for Eco-Environmental Sciences, Chinese Academy of Sciences, Beijing 100085, China; E-Mails: shipeng015@163.com (P.S.); xiaojuncas@gmail.com (J.X.); yfwang@rcees.ac.cn (Y.W.); 2 University of Chinese Academy of Sciences, Beijing 100049, China

**Keywords:** heavy metal, pipeline construction, risk assessment, spatial variation

## Abstract

The construction of large-scale infrastructures such as nature gas/oil pipelines involves extensive disturbance to regional ecosystems. Few studies have documented the soil degradation and heavy metal contamination caused by pipeline construction. In this study, chromium (Cr), cadmium (Cd), copper (Cu), nickel (Ni), lead (Pb) and zinc (Zn) levels were evaluated using Index of Geo-accumulation (*I_geo_*) and Potential Ecological Risk Index (RI) values, and human health risk assessments were used to elucidate the level and spatial variation of heavy metal pollution risks. The results showed that the impact zone of pipeline installation on soil heavy metal contamination was restricted to pipeline right-of-way (RoW), which had higher *I_geo_* of Cd, Cu, Ni and Pb than that of 20 m and 50 m. RI showed a declining tendency in different zones as follows: trench > working zone > piling area > 20 m > 50 m. Pipeline RoW resulted in higher human health risks than that of 20 m and 50 m, and children were more susceptible to non-carcinogenic hazard risk. Cluster analysis showed that Cu, Ni, Pb and Cd had similar sources, drawing attention to the anthropogenic activity. The findings in this study should help better understand the type, degree, scope and sources of heavy metal pollution from pipeline construction to reduce pollutant emissions, and are helpful in providing a scientific basis for future risk management.

## 1. Introduction

The ecological impact by linear projects is often the focus of attention in Environmental Impact Assessments (EIAs). Inevitably, natural gas/oil pipeline installation involves extensive disturbance to ecologic systems such as clearance of vegetation, ground excavation and soil compaction [1]. However, there has been little research describing soil heavy metal contamination due to the pipeline construction, and this may result in problems for EIA practitioners who might want to make quantitative assessments involving these activities. 

The serious concerns about increased heavy metal contamination in agricultural soils are well documented by the increasing number of papers on the subject in recent years. This increase is probably due to metal accumulation in the biota and their toxicity which can harm public health. It is widely recognized that intake of heavy metals via the soil-crop-human or soil-crop-animal-human chains are the predominant pathways of human exposure to environmental contamination in agricultural areas [2]. As many studies have reported, the main potential pollution sources of heavy metals in agricultural areas are derived from anthropogenic sources *i.e.*, fertilization, industrial wastes, irrigation using sewage, traffic, * etc*. [3,4,5]. However, large-scale infrastructure construction involves extensive anthropogenic activities and may be another important source of soil heavy metals, resulting in serious environmental problems, especially when the projects pass through agricultural areas. 

Pipeline right-of-way (RoW) construction involves different anthropogenic activities, including trenches, piling areas and working zones. Trenching and welding operations in the middle of pipeline RoW (trench area) may be the most important potential sources of heavy metal contamination. Discharges of heavy metals from vehicle traffic during the construction period and maintenance activities may be other important sources of metal pollution. The construction materials stacked in the piling area increase the risk of heavy metal contamination. It is necessary to clarify the spatial variation of heavy metal contamination from pipeline construction in farmland areas, considering that heavy metals may be introduced into the food chain, increasing health risks for the public from exposure to environmental heavy metals. However, the quantitative research on heavy metal contamination in different pipeline zones has not yet been reported.

A national network of pipelines spanning east to west and north to south has emerged in China, amounting to some 85,000 km at the end of 2010, and the total span will reach to 150,000 km by the end of 2015. Pipeline projects in China, taking the West-to-East Gas Pipeline as an example, have trenched through a number of farmlands. Heavy metal pollution in agricultural soils after the construction should be a matter of concern due to the potential health risks associated with the bio-concentration of non-biodegradable heavy metals [6]. In this study, the objectives were: (1) to identify whether pipeline construction causes soil heavy metal contamination; (2) to investigate the type of soil heavy metal contamination and estimate its spatial variation using two quantitative indices, the Index of Geo-accumulation (*I_geo_*) and the Potential Ecological Risk Index (*RI*); (3) to assess carcinogenic and non-carcinogenic health risks of soils polluted by pipeline construction activities. The results of this study will provide a scientific reference for decision makers to minimize the environmental risk of pipeline projects.

## 2. Experimental Section

### 2.1. Pipeline Introduction

**Figure 1:**
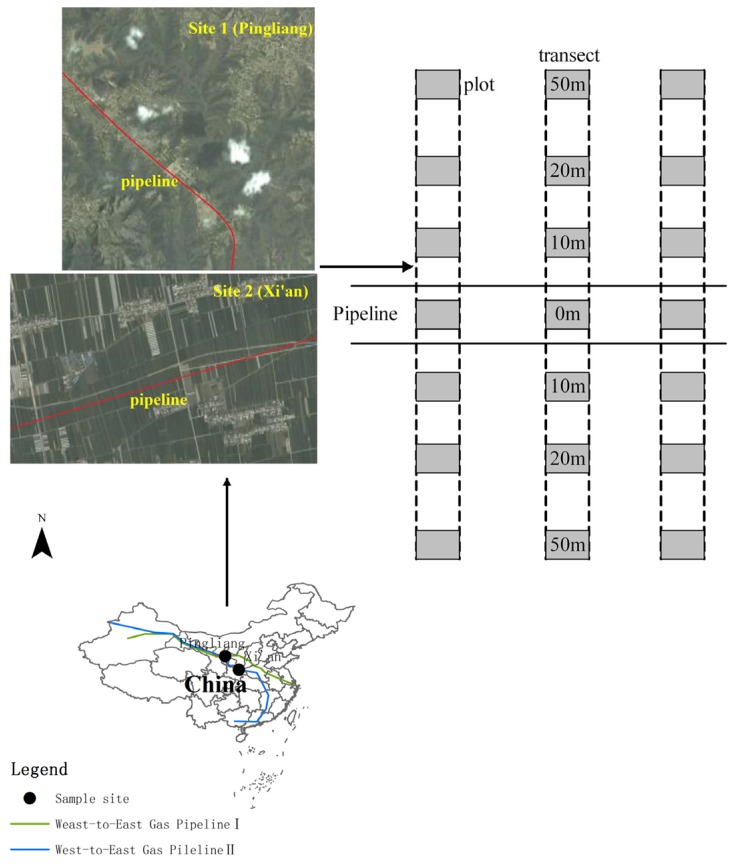
The two sample sites Site 1 (Pingliang) and Site 2 (Xi’an) along nature gas pipelines right-of-way and sample plots that were set up at seven different distances from the pipeline.

The nearly 4,000 km-long West-to-East Gas Pipeline I (WEGP I), with a diameter of 1,016 mm passes through Xinjiang, Gansu, Ningxia, Shaanxi, Shanxi, Henan, Anhui, and Jiangsu and terminates in Shanghai (Figure 1). It was put into operation in 2004. It then took three years from 2008 to 2010 to build the 4,843 km-long West-to-East Gas Pipeline II (WEGP II), running from northwestern Xinjiang to Guangzhou in Guangdong Province.

### 2.2. The Study Area

Two sites were set up in the typical northwest agricultural region of China including Pingliang (Site 1) and Xi’an (Site 2) (Figure 1). WEGP I and WEGP II pass through Site 1 and Site 2, respectively. Pipeline RoWs at both sites are located in crop fields. Site 1 is mainly derived from loess with texture ranging from fine to silt (with a 19.1% soil clay content) and the mean soil organic matter is 11.02 g/kg. Soil at site 2 is sandy loam textured (with a 22.9% soil clay content) and the mean soil organic matter is 17.11 g/kg. Soil pH values at the two sites range from 8.17 to 8.67.

### 2.3. Sample Methods and Laboratory Analysis

**Figure 2:**
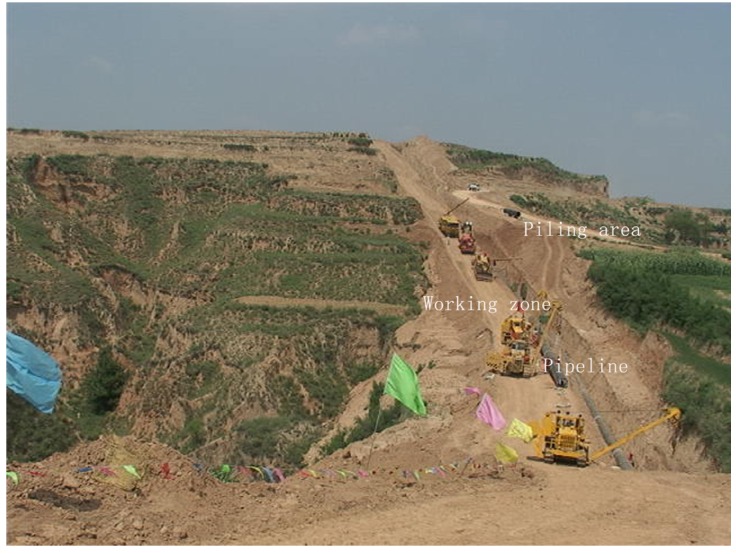
Pipeline right-of-way including trench, piling area and working zone.

Pipeline RoW of 28 to 30 m was divided into three zones (Figure 2): a trench area where the pipeline was buried (2 m width in the middle of pipeline RoW); a working zone used for vehicle movement (at one side of 2 to 15 m away from the trench); a piling area that was cleared to stockpile topsoil and subsoil stripped from the pipeline RoW (at other side 2 to 15 m away from the trench). In this study, three transects with similar topography and soil characteristics were set up at each site in Aug 2012, at more than 500 m distance away from each other (Figure 1). Each transect was perpendicular to the pipeline. Soils were sampled within the plots along transects at seven distances of 0 m (trench), 10 m (working zone or piling area), 20 m (E20 *vs*. W20 at Site 1 or N20 *vs*. S20 at Site 2), 50 m (E50 *vs*. W50 at Site 1 or N50 *vs*. S50 at Site 2) along both sides of pipeline. A plot of 1 m × 1 m in size was laid out for sampling along each transect at the aforementioned distances. Five randomly selected cores were taken to 10 cm depth from each plot to comprise a sample. For analysis of soil chemical properties, soil samples were air-dried at room temperature and sieved through 2-mm and 0.16-mm nylon sieves to remove coarse debris.

Soil pH was measured at a soil-to-water ratio of 1:2.5 using a Crison GLP 21 pH meter (Crison Instruments, Barcelona, Spain). Soil organic matter was determined using colorimetric determination [7]. Soil particle size was analyzed using the MasterSizer 2000 apparatus (Malvern Instruments Ltd, Malvern, UK). For the purposes of determining the total concentrations of Cd, Cr, Cu, Ni, Pb and Zn, soil samples were digested by HCl-HNO_3_-HF mixture in Teflon tubes using a MARS^TM^ Digestion & Extraction System (CEM, Boston, MA, USA). The presence of Cd and Ni in the extracts were detected using an Agilent 7500 inductively coupled plasma mass spectrometer (ICP-MS, Agilent Technologies, Santa Clara, CA, USA), and concentrations of Cr, Cu, Pb and Zn were analyzed using an inductively coupled plasma-optical emission spectrometer (ICP-OES, Agilent Technologies, Santa Clara, CA, USA). We used Chinese standard soil (GSS-8) to monitor the accuracy of the analytical procedure, and the quality control (QA/AC) data for the Cd, Cr, Cu, Ni, Pb and Zn measurements was about 105%. 

### 2.4. Data Analysis and Evaluation Methods

SPSS 16.0^®^ (IBM, Armonk, NY, USA) was used for statistical analysis. We compared means of heavy metal concentrations and soil contamination indices using one-way ANOVA analysis. Two contamination indices, namely the Index of Geo-accumulation (*I_geo_*) and the Potential Ecological Risk Index (*RI*) were calculated to evaluate soil heavy metal contamination. 

#### 2.4.1. Index of Geo-accumulation (*I_geo_*)

*I_geo_* was introduced by Müller [8] to assess metal pollution in sediments and has been applied in recent pollution studies to enable the qualitative assessment of soil contamination by heavy metals [9,10]. *I_geo_* is computed by Equation (1):

*I_geo _=* log_2 _(*C_n _/*1.5*B_n_*) (1)

where *C_n_* is the concentration of the element in the tested soil, *B_n_* is the geochemical background value in the average shale of element [11] and the constant 1.5 compensates for natural fluctuations of a given metal and for minor anthropogenic impacts [12]. The seven classes of *I_geo_* as proposed by Müller are as follows: *I_geo_* ≤ 0, uncontaminated (Class 0); 0 < *I_geo_* ≤ 1, uncontaminated to moderately contaminated (Class 1); 1 < *I_geo_* ≤ 2, moderately contaminated (Class 2); 2 < *I_geo_* ≤ 3, moderately to heavily contaminated (Class 3); 3 < *I_geo_* ≤ 4, heavily contaminated (Class 4); 4 < *I_geo_* ≤ 5, heavily to extremely contaminated (Class 5); *I_geo_* > 5, extremely contaminated (Class 6) [8]. 

#### 2.4.2. The Potential Ecological Risk Index (RI)

RI advanced by Hakanson [13], represents the toxicity of heavy metals and the response of the environment:

* RI = **∑** E_i_* (2)

*E_i _= T_i _f_i_* (3)

*f_ i_ = C_i_/B_i_* (4)

where RI is calculated as the sum of all six risk factors for heavy metals (Cd, Cr, Cu, Ni, Pb and Zn) in soils, *E_i_* is the monomial potential ecological risk factor, *T_i_* is the developed metal toxicity factor. The heavy metal toxicity is classified in the order of Cd > Cu = Ni = Pb > Cr > Zn. The toxic factors for Cu, Ni and Pb are 5, 30 for Cd, 2 for Cr and 1 for Zn, respectively. *f_i_* is the metal pollution factor, *C_i_* is the practical concentration of metals in soil, and *B_i_* is the background value for metals. The adjusted evaluation criteria for the potential ecological risk index were RI ≤ 50, low pollution; 50 < RI ≤ 100, moderate pollution; 100 < RI ≤ 200, considerable pollution; RI > 200, high pollution.

#### 2.4.3. Human Health Risk Assessment

Direct exposure of human to heavy metals in agricultural soil can occur through three exposure pathways [14]: (1) direct ingestion of soil; (2) inhalation of particulates emitted from soil; and (3) dermal absorption of heavy metals through soil adhered to exposed skin. Considering the different adverse health effects of heavy metals on humans, the corresponding non-carcinogenic and carcinogenic risks were calculated, adapted from USDOE [15] and USEPA [16].

Separately taking care of the hazard exposure for children and adults, non-carcinogenic hazard risk (HI) was calculated using Equations (5)–(9): 


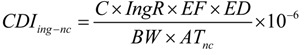
(5)


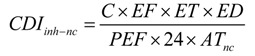
(6)


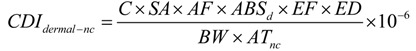
(7)


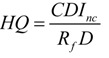
(8)


HI = **∑ ***HQ = HQ_ing_ + HQ_inh_ + HQ_dermal_* (9)

As carcinogenic substances, Cd, Cr, Ni and Pb were selected to assess the carcinogenic hazard risk (Total Risk) using Equations (10)–(16):

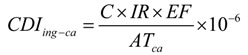
(10)

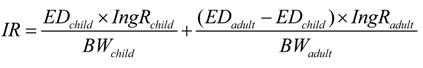
(11)

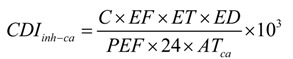
(12)

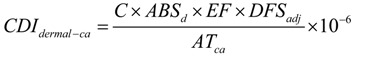
(13)

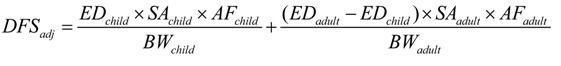
(14)

* Risk = CDI_ca_**×** CSF* (15)

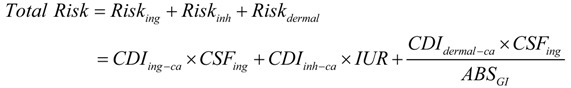
(16)
where *CDI_ing_*, *CDI_inh_*, *CDI_dermal_* were the chronic daily intake or dose contacted through oral ingestion (mg/kg/d), inhalation (mg/m^3^ for non-cancer and μg/m^3^ for cancer) and dermal contact with soil particles (mg/kg/d), respectively. *RfD* was the reference dose, and *RfD_ing_* (chronic oral reference dose), *RfC_inh _* (chronic inhalation reference concentration), *RdD_dermal_* (chronic dermal reference dose, *RfD_ing_* × *ABS_GI_*) via three exposure routes. *CSF* was the chronic slope factor, including *CSF_ing_*, *IUR*, *CSF_dermal_* for different exposure pathways. *CSF_dermal _*was calculated by *CSF_ing_*/*ABS_GI_*. The definitions and values of other parameters are shown in Table 1 and Table 2.

**Table 1:** Values of variables for human health risk assessment.

Parameters	Unit	Definition	Value		References
			Child	Adult	
C	mg/kg	heavy metal concentration			
ABS_d_	--	dermal absorption factor	0.03	0.001	[16]
AF	mg/cm^2^	soil to skin adherence factor	0.2	0.07	[15]
BW	kg	average body weight	16.2	61.8	[17]
ED	year	exposure duration	6	30	[15]
EF	d/year	exposure frequency	350	350	[15]
ET	h/d	exposure time	24	24	[15]
IngR	mg/d	soil ingestion rate for receptor	200	100	[15]
SA	cm^2^/event	Skin surface area available for exposure	2,800	5,700	[15]
AT_nc_	d	averaging time for non-carcinogenic	ED × 365		[15]
AT_ca_	d	averaging time for carcinogenic	LT × 365		[15]
DFS_adj_	mg × year/kg/d	soil dermal contact factor-age-adjusted	362.4		[18]
IR	mg × year/kg/d	Soil ingestion rate-age adjusted	113		[18]
LT	year	lifetime	72		[19]
PEF	m^3^/kg	Soil-to-air particulate emission factor	1.36 × 10^9^		[15]

**Table 2:** Toxicological parameters for different heavy metals of health risk assessment.

Elements	RfD_ing_ (mg/kg/d)	RfC_inh_ (mg/m^3^)	ABS_GI_	CSF_ing_ (mg/kg/d)	IUR (μg/m^3^)
Cd	1.0 × 10^−3^	1.0 × 10^−5^	0.025	--	1.8 × 10^−3^
Cr	3.0 × 10^−3^	--	0.013	--	1.2 × 10^−2^
Cu	4.0 × 10^−2^	--	1	--	--
Ni	2.0 × 10^−2^	9.0 × 10^−5^	0.04	--	2.6 × 10^−4^
Pb	3.5 × 10^−3^	--	1	8.5 × 10^−3^	1.2 × 10^−5^
Zn	3.0 × 10^−1^	--	1	--	--
References	[15]	[15]	[16]	[15]	[15]

## 3. Results and Discussion

### 3.1. Concentrations of Soil Heavy Metals

According to the Chinese Environmental Quality Standard, which is considerably higher than the corresponding natural background value of soils in China, Grade I was defined as natural soils without contamination (Table 3). Compared with the Chinese Environmental Quality Standard, it was shown that Cd, Cu, Ni in the trench area at Site 1 were over Grade I. Cd, Cu, Ni in trench and Cd, Pb in working zone, as well as Cu, Ni in the piling area at Site 2 exceeded Grade I; . It is thus indicated that pipeline construction may be a source of Cd, Cu, Ni and Pb pollution.

**Table 3:** Background values of heavy metal concentrations in the soils of the study areas.

Sites	Elements (mg/kg)					
	Cd	Cr	Cu	Ni	Pb	Zn
Site 1	0.107	57.5	23	29.3	16.8	67.9
Site 2	0.092	64.5	22.4	26.5	25.1	71.1
Standard ^a^	0.2	90	35	40	35	100

^a^ Chinese Environmental Quality Standard, Grade I [20].

**Figure 3:**
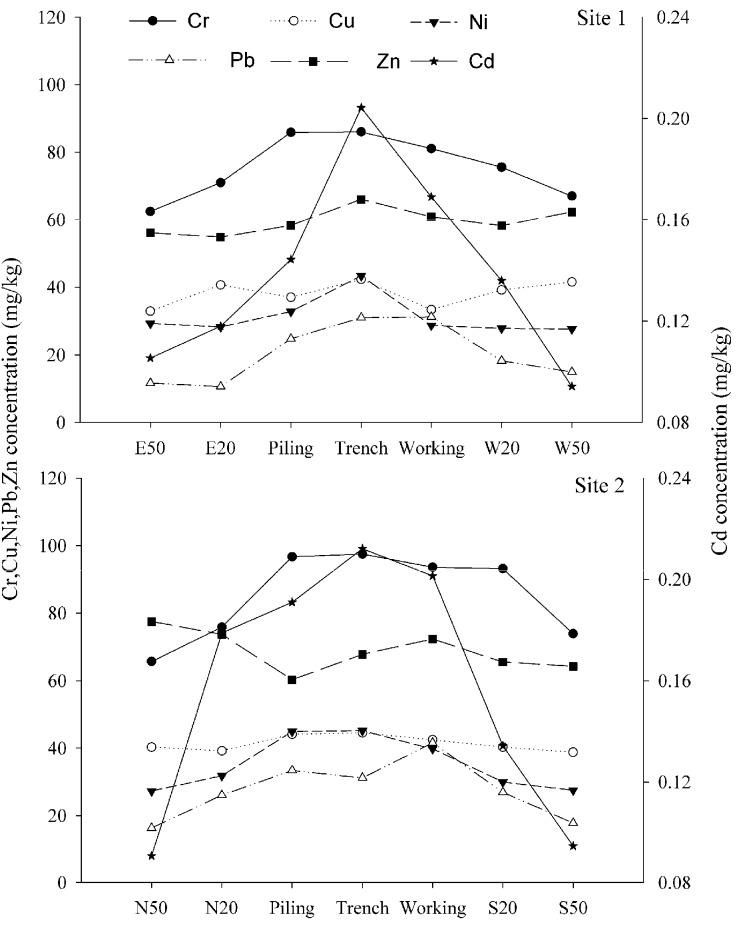
Concentrations of soil heavy metal at two sampling sites along pipeline. E: east, W: west, N: north, S: south.

Pipeline RoW including trenches, working zones and piling areas had higher concentrations of Cd, Cr, Ni, Pb and Zn than those at 20 m (E20 and W20) and 50 m (E50 and W50) distance from the pipeline at Site 1 (Figure 3). The maximum concentrations of Cd, Cu, Ni and Zn existed simultaneously in the samples collected from the trench at Site 1, and concentrations of Cd, Cu and Ni of trench were almost twice that of the background values. The highest concentration of 31.24 mg/kg for Pb was found in the working zone at Site 1 that was twice the background value (16.8 mg/kg). Cu, Ni, Pb, Zn concentrations were similar between 20 m and 50 m distances from both sides of the pipeline. Concentrations of Cd, Cr, Ni, Pb and Zn at 50 m distance from the pipeline were similar to background values at Site 1. There were increasing trends in the concentrations of Cd, Ni and Pb at Site 2 as follows: pipeline RoW zones (trench, piling area and working zone) > 20 m (N20 and S20) > 50 m (N50 and S50). The concentration of Pb at Site 2 was the highest in the working zone, followed by the trench and piling areas, respectively. Concentrations of Cd, Cr, Ni, Pb and Zn at 50 m distance from the pipelines were similar to the background values. The results indicated that the scale of the impact of pipeline construction on soil heavy metal contamination is restricted to some 20 m from the pipeline. 

### 3.2. Index of Geo-accumulation (I_geo_)

Pollution in agricultural areas exhibits highly adverse effects on humans, that is worthy of monitoring to evaluate their degree of pollution.

#### 3.2.1. Cadmium

In general, Cd, which exhibits highly adverse effects on human health, is considered one of the most eco-toxic metals. The highest value for *I_geo_* of Cd appeared in the trench area. The *I_geo_* obtained for the trench at Site 1 was 0.25, falling in Class 1 (uncontaminated to moderately contaminated, Table 4), while the value for the same pipeline zone of Site 2 was 1.31, classifying this soil as moderately contaminated (Table 5). Cadmium concentration in the topsoil of trench areas could be attributed to pipeline welding activities. Contaminated construction materials may be another source of Cd pollution in the pipeline RoW. The values of piling areas, working zones, N20, S20 at Site 2 and working zone at Site 1 ranged from 0.02 to 0.54, pointing to uncontaminated to moderately contaminated levels. The pollution sources of Cd in these areas mainly come from vehicles. The values of 50 m from both sides of pipeline at both of Site 1 and 2 were all measured at lower than 0, falling into Class 0, uncontaminated.

**Table 4:** Index of Geo-Accumulation in Pingliang (Site 1).

Pipeline zones	Cr	Cd	Cu	Ni	Pb	Zn
E50	−0.466	−0.608	−0.068	−0.646	−1.119	−0.864
E20	−0.282	−0.784	−0.020	−0.660	−1.371	−0.915
Piling	−0.006 ^*^	−0.170	0.098	−0.488	−0.133	−0.852
Trench	−0.004 ^*^	0.245	0.272	−0.123	0.291 ^*^	−0.690
Working	−0.091 ^*^	0.057	0.032	−0.639	0.235 ^*^	−0.765
W20	−0.192	−0.263	−0.003	−0.695	−0.487	−0.835
W50	−0.365	−0.870	0.146	−0.678	−0.801	−0.732

^*^ Significantly different at the 0.05 level.

**Table 5:** Index of geo-accumulation in Xi’an (Site 2).

Pipeline Zones	Cr	Cd	Cu	Ni	Pb	Zn
N50	−0.562	−0.631	0.260	−0.604	−1.218	−0.462
N20	−0.351	0.350	0.220	−0.349	−0.533	−0.544
Piling	−0.006 ^*^	0.460	0.384	0.150	−0.262	−0.839
Trench	0.004 ^*^	1.312 ^*^	0.304	−0.096	−0.343	−0.716
Working	−0.110 ^*^	0.544	0.334	−0.035	0.048	−0.634
S20	−0.054 ^*^	0.025	0.262	−0.115	−0.493	−0.704
S50	−0.390	−0.594	0.193	−0.341	−1.083	−0.733

^*^ Significantly different at the 0.05 level.

#### 3.2.2. Chromium

Cr is a low mobility element under moderately oxidizing conditions that is detrimental to biota [21]. Although Pipeline RoW had a higher *I_geo_* of Cr (*p* < 0.05) than that of 50 m distance from the pipeline on both sites, all the samples examined fell into Class 0, practically uncontaminated with Cr, as per Müller’s seven classes of *I_geo_*. It is indicated that pipeline construction may not cause Cr pollution. 

#### 3.2.3. Copper

Cu is considered as a micronutrient for plants and animals [22]. However, excess Cu in soils will induce stress and injury to plants [23]. The values of Cu at Site 1 ranged from a maximum of 0.27 for the trench area to a minimum of −0.07 at E50. Nearly all the samples at Site 1 and Site 2 fell into Class 1, uncontaminated to moderately contaminated. Pipeline RoW had a higher *I_geo_* for Cu compared to 20 m and 50 m distances from the pipeline at Site 2. Brake wear emissions from traffic in the working zone could be the main source of Cu. Gon [24] has reported that car wear, including brakes and tyres, was responsible for about 50% of the total copper emissions from road transport. 

#### 3.2.4. Nickel

Ni is a transition metal, and in recent years the concentration of Ni in the environment has been increasing due to human activities [25]. All the values obtained for Ni at Site 1 were negative, making it uncontaminated. The *I_geo_* was higher in the pipeline RoW at Site 1 compared to that of 20 m and 50 m distances from the pipeline, with the highest value in the trench areas. The value at 20 m distance from the pipeline (E20 and W20) was similar to that at 50 m distance from the pipeline at Site 1. The highest *I_geo_* for Ni at Site 2 was the piling area with a value of 0.15, indicating uncontaminated to moderately contaminated. All the other pipeline zones were negative, falling into Class 0. Pipeline RoW at Site 2 showed an increased *I_geo_* for Ni, while the lowest value appeared at N50. Emissions from mechanical wear of vehicles and oil burning may be the main sources of Ni contamination in pipeline zones. 

#### 3.2.5. Lead

Pb affects several human organs (*i.e.*, kidneys or liver). It has been accepted that Pb pollution in soils is a serious environmental problem [11]. The *I_geo_* for Pb in the trench area and working zone at Site 1 denoted an uncontaminated to moderately contaminated status, with values of 0.29 and 0.23, respectively. The value at the working zone of Site 2 was 0.05, indicating uncontaminated to moderately contaminated as well. Pipeline RoW showed increased values compared to 20 m and 50 m from the pipeline. Traffic is one of the major sources for Pb pollution [26]. Pb is widely used as an intermediate for antiknock additives for motor fuels, introducing lead into atmosphere and accumulating in the topsoil of roadside zone [27]. Farmlands on the roadside are vulnerable to be polluted by Pb because of oil consumption [28]. The policy of using lead-free fuel oil in China was not implemented until 2005, after construction of WEGP I and before WEGP II, and that may be the reason for the more serious contamination of Pb in the trench areas and working zones along WEGP I than for WEGP II. The values of N20 and S20 were higher than that of 50 m from the pipeline and all the *I_geo_* values at 20 m and 50 m were negative, indicating uncontaminated. Pb contamination decreased with distance from the pipeline, consistent with the results reported by Othman [29], who found that the contamination of Pb in the soil decreased with distance from the road, reaching background levels at 50 m from the road curb. 

#### 3.2.6. Zinc

There is a growing concern about zinc being one of the most readily mobile elements and the fact high doses of zinc will cause toxic and carcinogenic effects [10]. Collectively, the *I_geo_* values for Zn at both sites were all negative, revealing that all samples examined fell into Class 0. The highest value appeared in the trench area of Site 1, whilst the lowest one was for E20. The value at W50 was higher than that at 10 m and 20 m distances from the pipeline. In contrast, the highest value at Site 2 appeared at N50, whilst the lowest one happened in the piling area. Meanwhile, the values appeared higher at 20 m and 50 m from the pipeline than that at the trench area, indicating possible natural soil variability. It is therefore suggested that pipeline construction may not be the anthropogenic source of zinc pollution.

Generally, pipeline RoW had higher *I_geo_* values for Cd, Cr, Cu, Ni, Pb than those of 20 m and 50 m distances from the pipeline. The heavy metal pollution was slight at 50 m distance from the pipeline where the *I_geo_* values of Cr, Cd, Ni and Pb were negative, showing uncontaminated status.

### 3.3. Potential Ecological Risk Assessment

**Figure 4:**
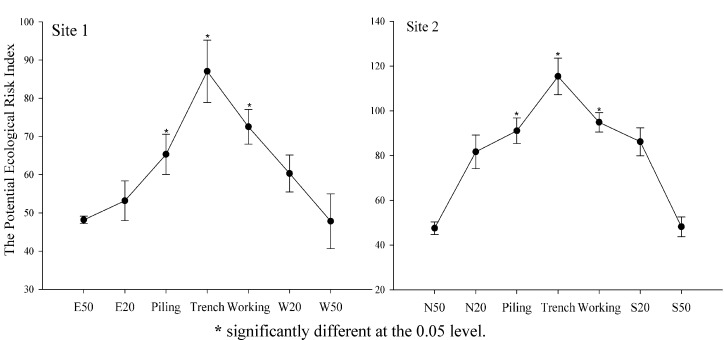
The Potential Ecological Risk Index at the two sampling sites alone pipelines.

It is well documented that the presence of highly toxic heavy metals can cause various types of health problems [30]. Excessive accumulation of heavy metals in agricultural soils will affect food quality and safety, increasing incidences of cancer, leukemia, reproductive disorders, and kidney or liver damage of people who are exposed to soil pollution [31]. To quantify the potential ecological hazard, the Potential Ecological Risk Index, RI, is calculated as the sum of all six heavy metals, which describes the sensitivity of the biological areas to the toxic heavy metals and illustrates the potential ecological risk caused by the pollution (Figure 4). RI results at both sites showed the same change tendencies within the different pipeline zones, in the sequence trench > working zone > piling area > 20 m zone > 50 m zone. The potential ecological risk of heavy metal pollution decreased with increasing distance from the pipeline, indicating that far away from the pipeline there was less soil contamination. Pipeline RoW at both Site 1 and 2 had much higher RI (*p* < 0.05) than that of 50 m distance from the pipeline. The trench area at Site 2 had a value of 115.4 for RI, showing considerable pollution, while the RI of the trench area at Site 1 was lower than 100, indicating moderate pollution. RI of the piling areas and working zones at both sites ranged from 65.3 to 91.1, showing moderate pollution. The higher potential ecological risk in the trench area, piling area and working zone could be attributed to emissions of heavy metals sourced from various anthropogenic activities that were mainly distributed along the pipeline RoW. The potential ecological risk at 20 m distance from the pipeline was moderate pollution, showing soil contaminations still existed at this distance. The values of RI at 50 m from both sides of the pipeline were lower than 50, showing a lower pollution. It is indicated that the impact of pipeline on soil pollution mainly occurred in the pipeline RoW and soils at 50 m distance from the pipeline are less polluted with heavy metals from the construction activities. 

* significantly different at the 0.05 level.

### 3.4. Human Health Risk Assessment

**Figure 5:**
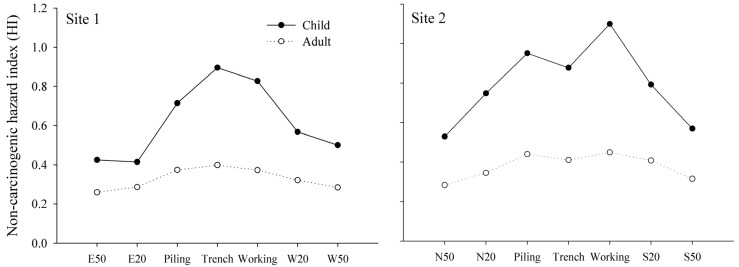
Non-carcinogenic hazard risk of children and adults at Sites 1 and 2 along the pipeline.

**Figure 6:**
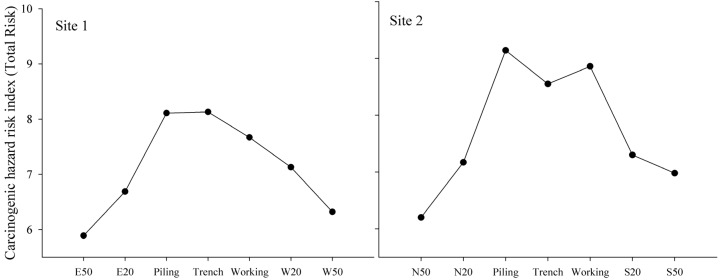
Carcinogenic hazard risk at Sites 1 and 2 along the pipelines.

Agriculture soil polluted with heavy metals can increase human health risks through different exposure pathways [32]. Inhalation of particulates, dermal contact and oral ingestion are considered as three main pathways for human exposure to soil heavy metals [18]. Non-carcinogenic hazard risk (HI) and carcinogenic hazard risk (Total Risk) in the pipeline RoW (piling, trench and working areas) were higher than those of 20 m and 50 m at both sites (Figure 5 and Figure 6), and can be attributed to pipeline construction. All operations were restricted to pipeline RoW (no more than 15 m away from one side of pipeline) according to the work regulations, which increased concentrations of soil heavy metal emitted from amounts of artificial activities in this area. The accumulation of soil toxics in pipeline RoW may form a pollution zone, and it will harm human health on a large scale. The carcinogenic hazard risk was higher at 20 m distance than that of 50 m distance, indicating that the risk of pipeline construction on human health was weakening, but still existed up to 20 m away from the pipelines. Non-carcinogenic hazard risk at both sites for children was relatively higher than for adults. This result was in agreement with Luo [18]. Children may absorb much more heavy metals from soils than adults during their outdoor play activities, resulting in more susceptibility for children to exposure to soil toxic metals [33]. Therefore, pipeline RoW was a high health hazard risk zone and children were more susceptible to non-carcinogenic hazard risks. The human health risk assessment is an effective approach to provide a quantitative determination for future risk management and environmental monitoring of pipeline construction. 

### 3.5. Relationships of Heavy Metals and Sources Analysis

**Figure 7:**
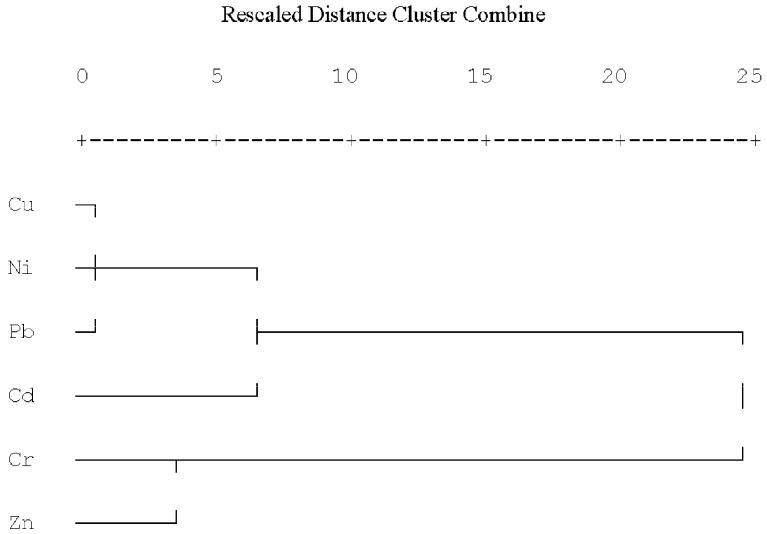
Hierarchical dendogram for six elements showing data clustering of variables.

Cluster analysis (CA) was used to identify the relationship among the analyzed elements and group their possible sources [34]. The distance cluster represented the degree of association between elements. The lower the distance cluster value, the more significant the relationship is [35]. The CA results were illustrated in a dendrogram, that grouped the six elements into two distinct clusters (Figure 7). The first cluster includes Cu, Ni, Pb and Cd. These metals draw attention to the anthropogenic sources which are associated with mechanical wear (*i.e.*, automobile tires and brake wear), welding (the incomplete cleanup of residual materials after welding) and burning of oil. Pipeline welding was the main source of Cd pollution in the trench area. Brake wear emissions from traffic increased Cu pollution in the working zone. Ni contamination in pipeline RoW may come from emissions due to mechanical wear and oil burning. Traffic in the pipeline construction is the main source for Pb pollution. The second cluster contained Cr and Zn, both of which are suggested as elements of a mixed origin. The results confirmed that soils in the pipeline zones were practically uncontaminated with Cr and Zn. It is thus indicated that emissions of Cr from the pipeline project are very low, and not enough to harm the environment. There was no clear change tendency for Zn at different distances from the pipeline. The main sources of Zn are not driven by the pipeline construction and fertilization maybe of greater contributor to Zn pollution in agricultural soils. 

It is worth paying more attention to heavy metal pollution caused by pipeline projects due to the fact heavy metals can be introduced into the food chain and thus threaten human health. For the purpose of minimizing this pollution, some necessary mitigation measures such as decreasing vehicle flow, using environment-friendly materials and banning littering should be implemented.

## 4. Conclusions

This study provides important information about the distribution of Cr, Cd, Cu, Ni, Pb and Zn in agricultural soils exposed to environmental stress by pipeline construction. Cluster analysis indicated that Cu, Ni, Pb and Cd had closer relationships, and their presence may be mainly related to vehicle emissions and welding during pipeline installation. The scale of soil heavy metal pollution due to pipeline construction was mainly restricted to the pipeline RoW. The RoW zones showed increased soil heavy metal concentrations, with higher *I_geo_* values for Cd, Cr, Ni, Pb than those at 20 m and 50 m distances from the pipeline. RI in these areas showed moderate to considerable pollution. Soil heavy metal pollution was slight at 50 m distance from the pipeline: concentrations of heavy metals detected at this distance were similar to background values and *I_geo_* values of Cd, Cr, Ni and Pb were negative showing uncontaminated status; the RI index showed a lower pollution. The human health risk assessment also showed that high risk zones were mainly in the pipeline RoW, and children were more susceptible to non-carcinogenic hazard risks. 

This study provides some early warning signals about soil heavy metal pollution from these types of project for EIA practice. Understanding the type, degree, scale and sources of heavy metal contaminations are essential for environmental management to reduce pollutant emission and minimize the hazard risks to human health. Furthermore, the results will help to design effective methods for successful soil restoration after pipeline construction.
